# Collateral hypersensitivity between ZY19489 and piperaquine neutralizes PfCRT-mediated drug efflux and Plasmodium falciparum resistance

**DOI:** 10.21203/rs.3.rs-7697517/v1

**Published:** 2025-10-09

**Authors:** John Okombo, Tolla Ndiaye, Tarrick Qahash, Igor M. R. Moura, Eva Gil-Iturbe, Laura M. Hagenah, Jessica L. Bridgford, Vinicius Bonatto, Kurt E. Ward, Tomas Yeo, Sunil K. Narwal, Lily V. Orta, Isla Anderson, Satish K. Dhingra, Charisse Flerida A. Pasaje, Heekuk Park, Jonathan Kim, Rafael V. C. Guido, Iñigo Angulo-Barturen, Jacquin C. Niles, Filippo Mancia, Anne-Catrin Uhlemann, Sachel Mok, Matthias Quick, Elizabeth A. Winzeler, Didier Leroy, Manuel Llinás, Vandana Thathy, David A. Fidock

**Affiliations:** 1 Department of Microbiology & Immunology, Columbia University Irving Medical Center, New York, NY, USA; 2 Center for Malaria Therapeutics and Antimicrobial Resistance, Division of Infectious Diseases, Department of Medicine, Columbia University Irving Medical Center, New York, NY, USA; 3 Department of Biochemistry & Molecular Biology, The Pennsylvania State University, University Park, PA, USA; 4 Huck Center for Malaria Research, The Pennsylvania State University, University Park, PA, USA; 5 São Carlos Institute of Physics, University of São Paulo, São Paulo, Brazil; 6 Department of Psychiatry, Columbia University Irving Medical Center, New York, NY, USA; 7 Department of Biological Engineering, Massachusetts Institute of Technology, Cambridge, MA, USA; 8 Division of Infectious Diseases, Department of Medicine, Columbia University Irving Medical Center, New York, NY, USA; 9 Department of Physiology and Cellular Biophysics, Columbia University Irving Medical Center, New York, NY, USA; 10 The Art of Discovery, Bizkaia 48160, Spain; 11 Department of Physiology and Cellular Biophysics, Columbia University Irving Medical Center, New York, NY, USA; 12 New York State Psychiatric Institute, Area Neuroscience – Molecular Therapeutics, New York, NY, USA; 13 Department of Pediatrics, School of Medicine, University of California, San Diego, CA, USA; 14 MMV Medicines for Malaria Venture, Geneva, Switzerland; 15 Department of Chemistry, The Pennsylvania State University, University Park, PA, USA.

## Abstract

New antimalarial drugs are essential to combat the current emergence and spread of *Plasmodium falciparum* parasite resistance to first-line artemisinin-based combination therapies. Here, we identify a mechanism of parasite resistance to ZY19489, a triaminopyrimidine currently in a Phase IIb clinical trial. Low-grade resistance was mediated by a novel mutation in the *P. falciparum* chloroquine resistance transporter PfCRT, which caused a major reduction in asexual blood stage parasite growth rates and a substantial fitness cost. Parasites resistant to ZY19489 lost their chloroquine resistance status and became hypersusceptible to the artemisinin-based combination partner drug piperaquine. All three agents were shown to interfere with parasite-mediated catabolism of host hemoglobin. Uptake studies in PfCRT-containing proteoliposomes provide evidence that ZY19489 can block mutant PfCRT-mediated efflux of piperaquine and chloroquine, creating a scenario of an evolutionary trap whereby resistance to ZY19489 blocks PfCRT efflux-mediated resistance and restores susceptibility to piperaquine and chloroquine. Metabolomic studies revealed that ZY19489 significantly reduces intracellular levels of short hemoglobin-derived peptides (a natural substrate of PfCRT) and leads to higher accumulation of pyrimidine deoxynucleotides. Our data present a marker for tracking the evolution of clinical resistance to ZY19489 and a rationale for pairing this with piperaquine to generate a novel resistance-refractory combination.

## Introduction

The World Health Organization recently reported an estimated 263 million cases and 597,000 malaria-related deaths globally in 2023^1^, highlighting the continuing upward trend in malaria infections since 2016. This upsurge in disease burden is multifactorial, compounded by the spread of *Plasmodium falciparum* alleles associated with loss of parasite sensitivity to artemisinin-based combination therapies (ACTs) and, more recently the emergence of parasites with deletions in histidine-rich proteins 2 and 3 (PfHRP2 and 3) that confound case detection^1–4^. Currently approved malaria vaccines, which offer moderate protection, are not yet widely available^5,6^. There is an urgent need for new, safe and efficacious antimalarial drugs endowed with distinct modes of action (MoA) to complement current treatment options.

**Fig. 1 |:**
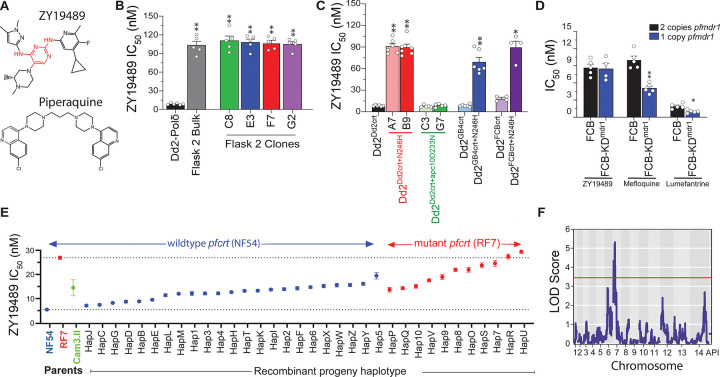
*Plasmodium falciparum* chloroquine resistance transporter (PfCRT) mediates *in vitro* resistance to ZY19489. **a** Chemical structures of ZY19489 and the 4-aminoquinoline, piperaquine (PPQ). **b** Drug susceptibility profiles are presented as mean ± SEM IC_50_ values of a ZY19489-selected bulk culture (red) and individual clones (C8, E3, F7 and G2) compared to their Dd2-Polδ parent (black). **c** Mean ± SEM IC_50_ values of *pfcrt*- and *pfapc10*-modified lines compared to their isogenic parents. Dd2^Dd2crt+N246H^ (red) and Dd2^Dd2crt+apc10 D233N^ (green) clones were compared to Dd2^Dd2crt^ (black) while Dd2^GB4crt+N246H^ (deep blue) and Dd2^FCBcrt+N246H^ (deep gray) were compared to Dd2^GB4crt^ (light blue) and Dd2^FCBcrt^ (light gray), respectively. **d** Mean ± SEM IC_50_ values for ZY19489, mefloquine (MFQ) and lumefantrine (LMF) against a FCB line (black) that expresses two copies of *pfmdr1* and the isogenic FCB-KD^*mdr1*^ (blue) line that expresses a single copy of the gene due to targeted disruption of the second (Supplementary Table 5). 72-hour susceptibility assays were performed in duplicate on 4 to 6 different occasions over a range of drug concentrations and statistical significance calculated using two-tailed Mann-Whitney *U* tests; *p < 0.05; **p < 0.01. **e** ZY19489 susceptibility of NF54 (blue), RF7 (red) and Cam3.II (green) parental lines and the 34 unique NF54×RF7 recombinant progeny with inherited NF54 wildtype or RF7 mutant *pfcrt* alleles. The dotted lines denote the baseline mean ZY19489 IC_50_ values against NF54 (5.8 nM) and RF7 (27.3 nM). **f** LOD (Logarithm of the Odds score) plots showing associations between ZY19489 IC_50_ values and segments across the *P. falciparum* genome. The red line signifies the 95% probability threshold. A locus on chromosome 7, which includes *pfcrt*, shows the highest association with ZY19489 *in vitro* activity.

Recent antimalarial drug discovery efforts aim to develop new medicines that overcome emerging drug resistance, whilst ensuring safety and improving dosing convenience. These efforts have yielded several new molecules with novel mechanistic profiles that are currently in various phases of clinical development (https://www.mmv.org/mmv-pipeline-antimalarial-drugs). One such candidate is ZY19489 (Fig. 1a), a fast-killing and long-acting triaminopyrimidine identified from a high-throughput screen of an AstraZeneca library comprising 500,000 compounds tested against *P. falciparum* asexual blood stage (ABS) parasites using high-content imaging^7^. This compound (also known as Zintrodiazine, MMV674253, AZ412, or TAP12) showed promising safety, pharmacokinetic, and efficacy results in a first-in-human clinical trial^8^. ZY19489 retains potency against field isolates expressing mutant *k13* (*Kelch13*) alleles that confer artemisinin partial resistance^9^. Currently, ZY19489 is in a Phase II trial in combination with ferroquine for the treatment of uncomplicated *P. falciparum* malaria (https://classic.clinicaltrials.gov/ct2/show/NCT05911828). If approved, this combination could constitute an important non-ACT treatment option in areas where artemisinin partial resistance has emerged^10^.

The putative MoA and molecular mechanisms that potentially underlie resistance to ZY19489 remain unknown. In a previous report, low-level resistance to this molecule was associated with a G29V mutation in subunit D of the *P. falciparum* vacuolar proton-transporting V-type ATPase complex (PfV1-D; PF3D7_1341900)^7^, suggesting possible involvement of this protein in conferring ZY19489 resistance or a direct target of the molecule. However, in a recent study using a “pH fingerprint” assay to elucidate the MoA of candidate antimalarials, ZY19489 did not affect the ability of the V-type H^+^ ATPase to regulate digestive vacuole (DV) or cytoplasmic pH, nor did it significantly inhibit the ATPase activity attributable to this pump^11^. These results indicate that the compound has a different MoA in the parasite. This obscure mechanistic and resistance profile subsequently limits rational selection of alternative partner molecules with which it could be paired for optimal clinical efficacy.

In this report, we explored the mechanistic and resistance features of ZY19489 by leveraging *in vitro* resistance selection and gene editing, genetic cross trait mapping, metabolomics, drug transport experiments, parasite growth fitness assays, and molecular dynamics simulations. This study identified a novel point mutation in the *P. falciparum* chloroquine resistance transporter (PfCRT; PF3D7_0709000) that confers low-grade resistance to this agent. We provide evidence of a high resistance barrier for ZY19489 based on combination treatments in mice and highlight its efficacy against resistance-conferring mutant alleles currently circulating in the field. Metabolomic results offer a window into the mechanistic basis of ZY19489 anti-ABS potency. These findings present a reliable marker for tracking the evolution of clinical resistance to this antimalarial drug and a rationale for potential pairing of this compound with the 4-aminoquinoline piperaquine (PPQ; Fig. 1a) to generate a resistance-refractory combination.

## Results

### *In vitro* selection and genome sequencing identify mutant parasites with low-grade resistance to ZY19489

Applying a ramping *in vitro* selection method to *P. falciparum* Dd2 parasites, a previous study observed low-grade resistance after ~200 days of drug pressure^7^. Whole-genome sequence (WGS) analysis associated resistance with a G29V mutation in PfV1-D (subunit D of the *P. falciparum* vacuolar proton-transporting V-type ATPase complex). To test whether this association was causal, we generated a conditional knockdown (cKD) line, NF54^V1-D cKD^ (Supplementary Table 1; Supplementary Fig. 1a-c), with PfV1-D expression regulated by anhydrotetracycline (aTc). Assays with the control V-type ATPase inhibitor bafilomycin A showed the expected reduction in IC_50_ (the half-maximal growth inhibition concentration) in parasites with reduced expression of PfV1-D. In contrast, we observed no difference between ZY19489 activity against PfV1-D cKD parasites cultured in high or low concentrations of aTc and control lines under no aTc, further confirming no causal role for the V-type ATPase complex (Supplementary Table 2).

To identify a robust basis of *in vitro* resistance to ZY19489, we used a high parasite inoculum strategy and the hypermutable Dd2-Polδ line with a higher basal mutation rate^12^ (Supplementary Fig. 2). Parasites were intermittently pressured with 100 nM ZY19489, corresponding to 10× its IC_50_. Recrudescent parasites obtained from one of the three flasks after 136 days of selection pressure exhibited a 12-fold IC_50_ increase compared to Dd2-Polδ. Resistance was confirmed in parasites cloned by limiting dilution (Fig. 1b; Supplementary Table 3).

WGS analysis of the ZY19489-pressured clones identified single nucleotide polymorphisms (SNPs) in four different genes (Table 1). Of these, the N246H mutation in *pfcrt* and D233N in the anaphase-promoting complex subunit 10 (*pfapc10*; PF3D7_1217600) were present in all clones. Mutations in two other genes were observed in a single clone (C8; Table 1), which also showed amplification of an ~80 kb chromosome 5 segment spanning 15 genes including the *P. falciparum* multidrug resistance protein 1 (PfMDR1; PF3D7_0523000; Supplementary Table 4).

### Genetic validation confirms PfCRT N246H as the primary determinant of ZY19489 resistance

To test the role of *pfcrt* N246H and *pfapc10* D233N, we genetically engineered these mutations into ZY19489-sensitive lines (Supplementary Table 1). N246H was edited into *pfcrt* in recombinant Dd2 lines expressing either the dominant Southeast Asian (Dd2^Dd2crt^) or two African (Dd2^GB4crt^ and Dd2^FCBcrt^) alleles, while *pfapc10* D233N was edited into Dd2^Dd2crt^. Gene editing used zinc-finger nucleases for *pfcrt* ^13^ and a CRISPR/Cas9 system for *pfapc*^12^ (Supplementary Fig. 3a, b). Successful editing yielded Dd2^Dd2crt+N246H^, Dd2^GB4crt+N246H^, Dd2^FCBcrt+N246H^, and Dd2^Dd2crt+apc10 D233N^, as confirmed by Sanger sequencing. We observed an 11-, 8- and 5-fold increase in ZY19489 IC50 values in the gene-edited Dd2^Dd2crt+N246H^, Dd2^GB4crt+N246H^ and Dd2^FCBcrt+N246H^ lines, respectively, compared to their isogenic parental lines (Fig. 1c; Supplementary Table 5). These shifts reproducibly phenocopied the results from the drug-selected Dd2 clones, albeit with a marginally lower fold change in Dd2^GB4crt+N246H^ and Dd2^FCBcrt+N246H^. On the other hand, the PfAPC10 mutant clones (with mean ± SEM ZY19489 IC_50_; 7.7 ± 0.7 nM and 8.8 ± 1.0 nM) did not show any significant change in susceptibility compared to Dd2^Dd2crt^ (mean ± SEM IC_50_; 8.5 ± 0.4 nM) (Fig. 1c; Supplementary Table 5). Commercially sourced APC10 and ubiquitin ligase inhibitors tested against Dd2^Dd2crt+apc10 D233N^ and its isogenic parent showed no difference in activity (Supplementary Table 6), further arguing against a modulatory role for the *pfapc10* D233N mutation. The K242T and T1776I mutations observed in PF3D7_1320800 and PF3D7_1321300, respectively, were not studied further since they were present in only one clone.

Because the WGS analysis had also flagged copy number amplification on chromosome 5 that includes *pfmdr1*, we also examined the influence of differential copies of this pleotropic efflux transporter on ZY19489 activity by testing the susceptibility of two isogenic FCB lines that differ in their *pfmdr1* copy number and expression levels^14^. These lines showed no significant difference in susceptibility to ZY19489. The aryl amino alcohols, mefloquine and lumefantrine (Supplementary Fig. 4), both showed a ~2-fold IC_50_ decrease against the single-copy FCB-KD^*mdr1*^ relative to the parental line that expresses two copies, as expected^14^ (Fig. 1d; Supplementary Table 7). These findings establish the PfCRT N246H mutation as the primary driver of *in vitro* resistance to ZY19489.

### Quantitative trait loci analysis map *in vitro* ZY19489 resistance to a chromosome 7 segment containing *pfcrt*

We tested the activity of ZY19489 against contemporary Cambodian clinical isolates and observed 2.3- and 4.7-fold higher mean IC_50_ values against Cam3.II (14.9 ± 3.3 nM) and RF7 (27.3 ± 0.7 nM), respectively, compared to the drug-sensitive African reference line NF54 (5.8 ± 0.2 nM). This suggests the presence of naturally occurring field mutations (present in Cam3.II and RF7) that might modulate parasite response to this compound. We therefore leveraged a genetic cross conducted between the PPQ- and dihydroartemisinin-resistant RF7 and NF54 that was previously performed^15^, and performed quantitative trait locus (QTL) mapping to localize candidate determinants of ZY19489 susceptibility.

Phenotype profiling of the 34 recombinant progeny clones recovered from that cross revealed ZY19489 IC_50_ values ranging from 8 to 30 nM (Fig. 1e), which allowed for a phenotype-genotype linkage analysis to uncover QTLs associated with partial resistance to ZY19489. QTL analysis revealed a significant peak on chromosome 7 that spanned 300 kb and contained 34 genes with non-synonymous mutations between NF54 and RF7 (Supplementary Table 8). The maximum LOD (Logarithm of the Odds) score of 5.3 within this segment corresponded to *pfcrt*, suggesting a strong association between the *pfcrt* Dd2+M343L allele present in RF7 and ZY19489 *in vitro* activity (Fig. 1f). These observations, along with the WGS data from the *in vitro* selected clones and genetic validation experiments, confirm that PfCRT mutations can modulate ZY19489 *in vitro* activity.

### Structural evidence suggests that the N246H mutation alters ZY19489 interactions with the PfCRT central cavity

**Fig. 2 |:**
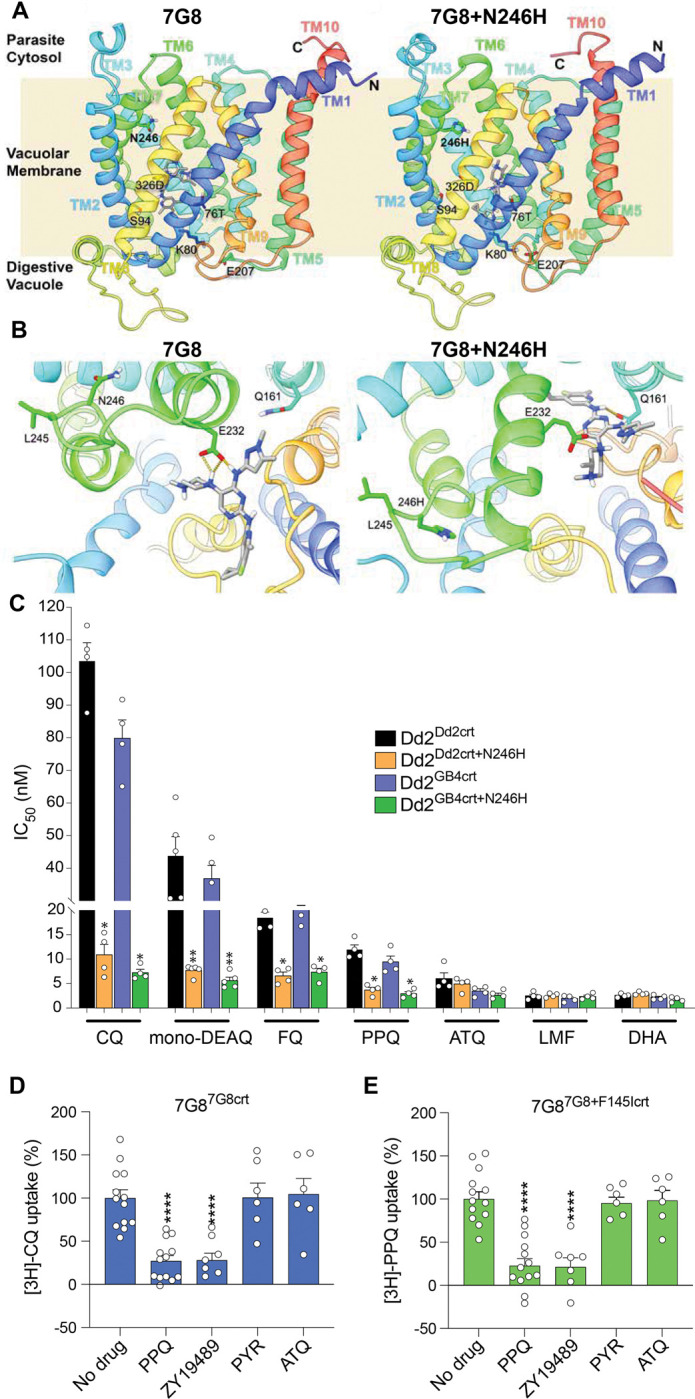
PfCRT interacts with ZY19489, with this drug interaction interfering with PfCRT-mediated CQ and PPQ efflux. **a** PfCRT in complex with ZY19489 (in gray and blue stick form) in the 7G8 wildtype (N246) and mutant (246H) protein. N246H localizes to transmembrane 7 (green) in the central cavity, close to the parasite cytosol. In the open-to-DV conformation, the ligand preferentially resides near the 76T region but does not interact with either N246 or 246H. **b** Hydrogen bonding patterns between ZY19489 and 7G8 PfCRT in the open-to-cytosol conformation implicate the side chain of E232 and the backbone carbonyl group of L245 as important for stabilizing drug-transporter interactions. Bonding pattern with 7G8+N246H PfCRT provides evidence that a single significant hydrogen bond persisted more than ~30% of the simulation time between the carbonyl oxygen of the Q161 side chain and the ligand. Hydrogen bonds are represented as dashed blue lines. **c** Parasite susceptibility profiles for chloroquine (CQ), mono-desethylamodiaquine (mono-DEAQ), ferroquine (FQ), piperaquine (PPQ), atovaquone (ATQ), lumefantrine (LMF), and dihydroartemisinin (DHA), tested against Dd2^Dd2crt^ (black), Dd2^Dd2crt+N246H^ (orange), Dd2^GB4crt^ (blue) and Dd2^GB4crt+N246H^ (green). Data show mean IC_50_ ± SEM values calculated from 4 to 6 independent experiments conducted in duplicates. Statistical significance was calculated using two-tailed Mann-Whitney *U* tests: *p < 0.05 and **p < 0.01. Uptake of 100 nM [^3^H]CQ (**d**) and [^3^H]PPQ (**e**) in proteoliposomes containing 7G8^7G8crt^ (**d**) and 7G8^7G8+F145Icrt^ (**e**) variants normalized to control liposomes. Data represent mean ± SEM of 6 to 13 independent experiments. In panels **d** and **e**, statistical significance was calculated using two-tailed Mann-Whitney *U* tests: ****p <0.0001.

PfCRT localizes to the ABS parasite’s digestive vacuole (DV) membrane and comprises 10 transmembrane (TM) helices, including four antiparallel pairs that surround a large central cavity^16^. Distinct amino acid substitutions, including those that confer chloroquine (CQ) or PPQ resistance, line this cavity and mediate the conversion of drug binding events into transport across the DV membrane^16^. To understand the effect of the N246H mutation on the PfCRT structure and elucidate the protein-ligand interactions that govern ZY19489 resistance, we performed atomistic molecular dynamics simulations with the cryo-EM elucidated 7G8 structure and a mutated version into which we introduced the N246H mutation. Our results show that the mutated 246H residue lies on TM7 and lines the central cavity close to the parasite cytosol (Fig. 2a). In the open-to-DV conformation, ZY19489 preferentially resides near the CQ resistance-conferring 76T residue in both the wildtype (7G8) and N246H-mutated proteins and has no direct interaction with residue 246 (Fig. 2a).With the 7G8 isoform, ZY19489 adopted orientations that facilitate hydrogen bonding with residues such as 326D or S94, unlike in the 7G8+N246H mutant where no specific ligand-protein hydrogen bonds persisted for more than 15% of the simulation time. This suggests a weaker interaction in the mutated protein than in 7G8. Additionally, ZY19489 disrupts the residue interaction network around 76T (Y68, S72, N75, 76T, H97, 326D, D329, Q352) more substantially in 7G8 than in the 7G8+N246H variant. Interestingly, the 7G8+N246H protein forms a salt bridge between K80 and E207, or other nearby residues, particularly in the regions between TM1-TM6, TM5-TM9, and TM6-TM9 (Supplementary Fig. 5). This salt bridge formation alludes to a tendency for the mutant protein to adopt a closed-to-DV (occluded) conformation and suggests that the mutation induces resistance to ZY19489 by favoring this closed state that may facilitate drug transport. Conversely, the 7G8 protein does not adopt this DV-closed conformation, potentially resulting in reduced transport activity (Supplementary Fig. 5).

In the open-to-cytosol model, we observed significant differences in the hydrogen bonding patterns, favoring stronger interactions and stabilization of ZY19489 with 7G8 than with the mutated protein. Specifically, hydrogen bonds with the side chain of E232 and the backbone carbonyl group of L245 appeared to be important for stabilizing the ligand in the 7G8 isoform (Fig. 2b). In the 7G8+N246H variant, we observed only one hydrogen bond interaction with Q161 that persisted for more than 30% of the simulation period. This absence of consistent hydrogen-bonding in the 7G8+N246H mutant points to weaker ligand interactions compared to the 7G8 isoform. These findings suggest that the N246H mutation may significantly alter the binding dynamics of ZY19489 in the open-to-cytosol conformation, reducing the interaction stability of the ligand and subsequently facilitating its transport in the mutated protein, leading to resistance.

### PfCRT N246H confers sensitivity to DV-acting aminoquinoline antimalarials

PfCRT mutations can mediate parasite sensitivity to 4-aminoquinoline ring-containing antimalarials whose activity can be impacted by pH changes in the DV^16,17^. To relate the molecular dynamics simulation results to the experimentally derived influence of PfCRT N246H on various antimalarials, we tested the susceptibility of Dd2^Dd2crt+N246H^, Dd2^FCBcrt+N246H^ and Dd2^GB4crt+N246H^ to clinically important drugs including dihydroartemisinin, CQ, ferroquine, atovaquone (ATQ), mono-desethylamodiaquine, PPQ and lumefantrine (Supplementary Fig. 4). This antimalarial catalogue was curated to comprise DV-acting drugs as well as inhibitors whose primary targets lie elsewhere. Dd2^Dd2crt+N246H^ and Dd2^GB4crt+N246H^ were 9- and 11-times significantly more susceptible to CQ, respectively, than their parental lines, consistent with ablation of CQ resistance (Fig. 2c; Supplementary Table 9). These lines also exhibited 2- to 3-fold sensitization to ferroquine and PPQ as well as a 4- to 6-fold increased sensitivity to mono-desethylamodiaquine. Although the same trend was also observed for lumefantrine, the drop in IC_50_ was modest and not statistically significant against Dd2^Dd2crt+N246H^ (IC50 of 4.9 nM *vs* Dd2^Dd2crt^ IC50 of 6.0 nM) or Dd2^GB4crt+N246H^ (IC50 of 2.8 nM *vs* Dd^2GB4crt^ IC_50_ of 3.4 nM). There was no significant change in susceptibility towards dihydroartemisinin and ATQ (Fig. 2c; Supplementary Table 9), alluding to a collateral sensitivity specific to DV-acting aminoquinolines.

### ZY19489 competes with CQ and PPQ in PfCRT-mediated drug efflux

To interrogate the PfCRT-mediated transport of ZY19489 across the DV membrane, we leveraged a drug transport model that mimics an “inside-out” parasite DV whereby [^3^H]CQ or [^3^H]PPQ uptake can be measured in PfCRT-containing proteoliposomes^16^. We observed a significant 72% reduction in [^3^H]CQ transport by the CQ-resistant 7G8 isoform upon addition of 1 μM ZY19489 (Mann-Whitney *U* tests; *p* < 0.0001), suggesting that this compound can directly compete with CQ for transport (Fig. 2d). Similar competition was also observed against [^3^H]PPQ transport by the PPQ-resistant isoform 7G8+F145I, with a significant 79% inhibition of transport achieved with 1 μM ZY19489 (Mann-Whitney *U* tests; *p* < 0.0001, Fig. 2e). Transport of [^3^H]CQ and [^3^H]PPQ by the PfCRT isoforms 7G8 and 7G8+F145I, respectively, were significantly inhibited by the addition of the reciprocal agents PPQ and CQ, to levels comparable to those observed with ZY19489 (Mann-Whitney *U* tests; *p* < 0.0001, Fig. 2d and 2e). No competitive inhibition of transport was observed with pyrimethamine or ATQ whose resistance mechanisms are unrelated to PfCRT-mediated transport. These results show that ZY19489 can antagonize CQ and PPQ transport and offer evidence of competition for the protein’s binding cavity. They also provide compelling evidence favoring ZY19489 as an agent that can restore CQ or PPQ potency by inhibiting their mutant PfCRT-mediated drug efflux.

### PfCRT N246H confers a slow growth rate and high fitness cost on ABS parasites

**Fig 3 |:**
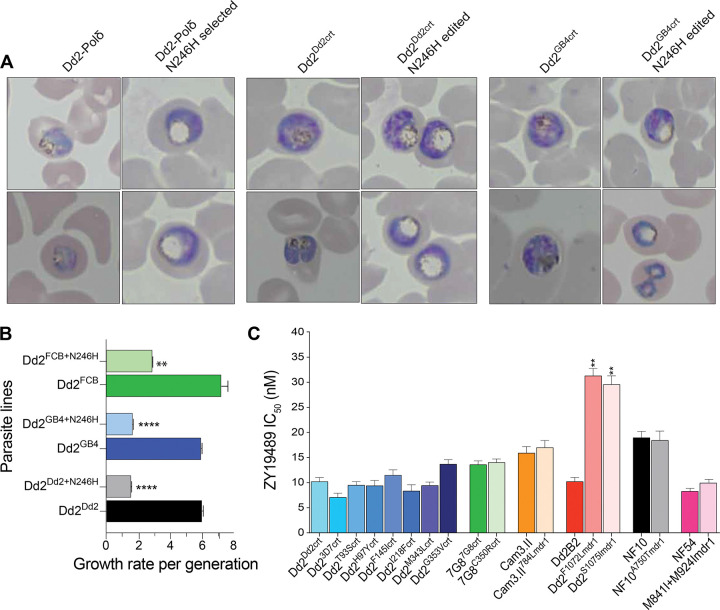
ZY19489 retains potency against parasites with contemporary *pfcrt* and *pfmdr1* mutations and parasites resistant to this compound display aberrant morphology and substantial fitness cost. **a** Light microscopy images reveal distended, translucent DVs in Dd2 parasites expressing the N246H mutation in three distinct CQ-resistant mutant *pfcrt* allelic backgrounds. Distended DVs were not observed in Dd2-Polδ, Dd2^Dd2crt^ or Dd2^GB4crt^ parental lines lacking the PfCRT N246H mutation. **b** *In vitro* growth characteristics of PfCRT N246H mutant lines and their isogenic parents. Mean ± SEM growth rates per generation were defined as the average rate of parasite growth per generation over eight generations, conducted on three separate occasions. Statistical significance was calculated using two-tailed Student’s *t* tests with Welch’s correction; **p < 0.01; ****p < 0.0001. **c** *In vitro* 72-hour activity of ZY19489 against naturally occurring or drug-selected contemporary *pfcrt* and *pfmdr1* alleles. The susceptibility of each mutant line was compared to their respective isogenic parental edited or unedited lines. Data are presented as mean ± SEM IC_50_ values calculated from 4 to 5 independent experiments conducted in duplicates. Statistical significance was calculated using two-tailed Mann-Whitney *U* tests; **p < 0.01.

During routine culturing of the ZY19489-pressured, resistant Dd2-Polδ mutants, we noticed a unique parasite morphology characterized by translucent distended DVs during the development from mid-trophozoites to mid-schizonts (Fig. 3a). This aberrant phenotype was absent in the Dd2-Polδ, Dd2^Dd2crt^ and N233D *pfapc10*-edited clones but was retained in all N246H *pfcrt*-edited lines, suggesting an interplay between this mutation and parasite DV physiology.

To assess the impact of this mutation on parasite growth rate, we monitored the parasite replication rates of synchronized ring stage parasites over several replication cycles and used it as a proxy for ABS fitness. Dd2^Dd2crt+N246H^, Dd2^GB4crt+N246H^, Dd2^FCBcrt+N246H^ and their parental lines were all seeded at an inoculum equivalent to 8×10^5^ parasites per well and were followed over 8 generations (16 days). Parasitemia measurements and media change were performed on day 3 and 4 and every two days thereafter. Parasite cultures with >2% parasitemia were diluted to 0.2% to reinitiate growth. Dd2^Dd2crt^, Dd2^FCBcrt^ and Dd2^GB4crt^ exhibited a 6- to 7-fold mean growth rate per generation compared to 2- to 3-fold rates observed in the mutants over the same period. From these data, we quantified the fitness cost of each mutant as a percent reduction in growth rate per generation relative to the isogenic parent (Fig. 3b). Dd2^Dd2crt+N246H^, Dd2^GB4crt+N246H^ and Dd2^FCBcrt+N246H^ exhibited a mean fitness cost of 75%, 73% and 61%, respectively, consistent with the slow expansion rates in routine cultures. These results suggest that PfCRT N246H confers a severe fitness cost to ABS parasites. We hypothesize that parasites with this allele would be likely to have a major competitive disadvantage were they to emerge in the field.

### ZY19489 retains potency against contemporary mutant *pfcrt* and *pfmdr1* alleles

The combination of ZY19489 and ferroquine has the potential to be a valuable non-ACT antimalarial therapy. We investigated whether parasites with contemporary *pfcrt* and *pfmdr1* alleles known to confer resistance to PPQ or other clinically relevant drugs could also be cross-resistant to ZY19489. Assays included CQ-resistant lines harboring different PfCRT mutations at residues 72–76 as well as PPQ-resistant lines expressing causal PfCRT mutations including C350R, H97Y, F145I, M343L, or G353V. We extended this screening to naturally occurring or drug-selected *pfmdr1* mutants on NF10, NF54, Cam3.II and Dd2 backgrounds (Supplementary Table 1). A750T and S784L are naturally occurring PfMDR1 mutations previously identified in field isolates from Western Cambodia and the Thai-Myanmar border^18,19^ while the M841I+M924I double mutant was obtained from *in vitro* drug selection using the piperazine-containing ACT-451840^20^. The F1072L and S1075I PfMDR1 mutants were obtained from selection with hexahydroquinoline derivatives^21^.

ZY19489 potency was equivalent against all CQ- or PPQ-resistant *pfcrt* mutant alleles expressed in the Dd2 parasite background, with mean IC_50_ values ranging from 7.1 nM (Dd2^3D7crt^) to 13.7 nM (Dd2^G353Vcrt^; Fig. 3c; Supplementary Table 10). Against CQ-resistant 7G8^7G8crt^ and PPQ-resistant 7G8^C350Rcrt^ parasites, the ZY19489 mean IC_50_ was 13.6 nM and 14 nM, respectively, highlighting the equipotency of this compound against mutant *pfcrt* alleles presently circulating in the field. The sensitivity of mutant PfMDR1 parasites, on the other hand, was more varied. There was no difference in potency against NF54 or NF54^M841I+M924Imdr1^ parasites (mean IC_50_s; 8.3 nM and 9.9 nM, respectively). Similarly, ZY19489 was equipotent against NF10^A750Tmdr1^ and Cam3.II C580Y^S784Lmdr1^ compared with their NF10 and Cam3.II C580Y parental lines. In contrast, we observed a 3-fold decrease in ZY19489 activity against Dd2^F1072Lmdr1^ (mean IC_50_; 31.3 nM) and Dd2^S10751mdr1^ (mean IC_50_; 29.6 nM) compared to the parental Dd2-B2 line (mean IC_50_; 10.2 nM) (Fig. 3c; Supplementary Table 10).

### ZY19489 exhibits a high *in vivo* barrier to resistance in mice treated in combination with other candidate antimalarials

To assess whether ZY19489-resistant parasites emerge *in vivo* following combination treatment, we used a chimeric humanized NOD-*scid IL-2Rγ*^*null*^ (NSG) mouse model of *P. falciparum* ABS infection^22^. In three independent experiments, humanized NSG mice infected with the 3D7^0087/N9^ strain were treated for 1 to 4 days with varying doses of ZY19489, the quinoline-carboxamide M5717^23^, the pantothenamide MMV693183^24^, or ZY19489-based combinations with MMV693183, ferroquine, or M5717 (Table 2).

In the ZY19489 + MMV693183 treatment group, 11 recrudescent parasite lines (10 from various dosing regimens of the combination and 1 from a 3-day MMV693183 monotherapy at 2.5 mg/kg) were culture-adapted for phenotypic screening and WGS. No significant differences in parasite *in vitro* responses to ZY19489 or MMV693183 were observed between the parental 3D7^0087/N9^ strain and any of the recrudescent lines (Table 2). WGS identified genetic changes in only one line, TAD_779, which carried a Y1020H mutation in PF3D7_0825000, encoding a conserved *Plasmodium* protein of unknown function, and a N636Y mutation in PF3D7_1416100, encoding SEY1, a putative endoplasmic reticulum-shaping protein. Neither mutation was associated with altered compound susceptibility. Notably, no mutations were observed in PF3D7_0627800 encoding the CoA-binding enzyme acetyl-CoA synthetase, a known resistance marker for MMV693183^24,25^. These findings suggest that recrudescence was likely due to sub-optimal drug exposure rather than acquired resistance to ZY19489 or MMV693183.

Similarly, no significant shifts in IC_50_ or IC_90_ values for ZY19489 or ferroquine were observed in lines recrudescing after exposure to 4 × 40 mg/kg ZY19489 alone or in combination with one dose of 15 mg/kg ferroquine (Table 2). WGS revealed no consistent mutations across ZY19489− or ZY19489+ferroquine-treated lines, indicating no evidence of acquired resistance to either compound.

In the group treated with either M5717 alone or in combination with ZY19489, seven recrudescent samples were obtained (three from M5717 monotherapy and four from combination regimens). These samples, along with the untreated parental strain, were not available for *in vitro* phenotypic testing but were genotyped at the *Pf*e*EF2* (PF3D7_1451100) locus that encodes *P. falciparum* translation eukaryotic elongation factor, a known target of M5717. Three lines, TAD_382, TAD_384, and TAD_400, harbored the *Pf*e*EF2* Y186N mutation, which confers high-grade M5717 resistance (Table 2)^23,26^. Two of these (TAD_382 and TAD_384) were from M5717 monotherapies; the third (TAD_400) emerged following 20 mg/kg ZY19489 + 20 mg/kg M5717 combination treatment. WGS was performed on five (TAD_382 and all four from the combination treatment) to identify additional mutations. The *Pf*eEF2 Y186N mutation was confirmed in TAD_382 and TAD_400, consistent with targeted amplicon sequencing. Three additional missense mutations were identified in TAD_382, including a P490L substitution in PF3D7_1032000 (ribosome maturation factor RimM), which was present in all five M5717-exposed lines (Table 2). However, no novel SNPs or CNVs were observed in samples exclusively exposed to the ZY19489+M5717 combination, confirming that *Pf*eEF2 Y186N likely arose from M5717 pressure.

To assess potential cross-resistance between M5717 and ZY19489, we tested three M5717-resistant lines (TAD_470, TAD_462, and TAD_464), which harbored distinct *Pf*eEF2 mutations (I183M, P754S, and I182T, respectively), for susceptibility to ZY19489. No significant differences in IC_50_ or IC_90_ values were observed compared with the drug-sensitive parental line (TAD_022), indicating that *Pf*eEF2-mediated M5717 resistance does not affect parasite sensitivity to ZY19489 (Supplementary Table 11). M5717 susceptibility testing confirmed the previously reported resistance phenotypes of these lines, with IC_50_ shifts of 4.5-fold (TAD_470), 151-fold (TAD_462), and ~260-fold (TAD_464) relative to TAD_022 (Supplementary Table 11)^26^. Together, these data underscore the high *in vivo* barrier to resistance against ZY19489.

### ZY19489 *in vitro* ABS potency is maximal against rings and schizonts

**Fig. 4 |:**
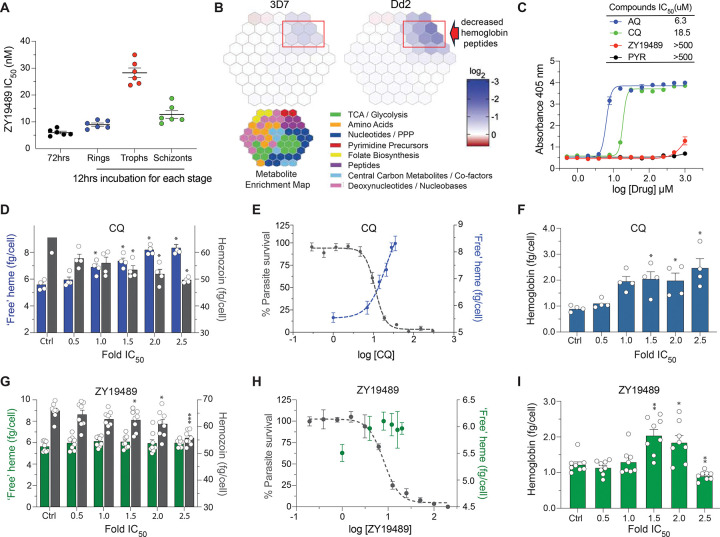
ZY19489 is active against rings and schizont and perturbs Hb catabolism: **a** Dot plots showing the stage-specificity profile of ZY19489 against synchronized 3D7 rings (blue), trophozoites (red) and schizonts (green) exposed to drug for 12 hours before drug wash-off. IC_50_ values were assessed at 60 hours and data are presented as means ± SEM from six independent experiments. **b** Metabolomic profiles associated with synchronized 3D7 and Dd2 trophozoites following treatment with 10× ZY19489 for 2.5 hours. Data are displayed as supra-hexagonal meta-prints determined from the log_2_ fold change values of targeted metabolites following drug treatment relative to no-drug control. Metabolite clusters were associated with eight generalized metabolic pathways using the KEGG database and color coded within the supra-hexagon base map. The red grids highlight the hexagon panels associated with Hb-derived peptides. **c** Plot of inhibition of β-hematin formation versus concentration profiles for ZY19489 relative to known antimalarial standards. Data were fitted to the sigmoidal concentration response (variable slope) equation in GraphPad Prism to determine the IC_50_, represented as the mean of four independent experiments performed in technical duplicates. (**d, f, g** and **i**) Heme fractionation of CQ- and ZY19489-treated NF54 parasites with different drug concentrations. Heme species are presented as ‘free’ heme (left y-axis) and hemozoin (right y-axis) Fe in femtogram isolated per cell (fg/cell) following exposure to different concentrations of (**d**) CQ or (**g**) ZY19489. Amounts of hemoglobin Fe (fg/cell) are shown in panels **f** and **i**. Data are presented as means ± SEM from 4 to 8 repeats and statistical comparisons of the drug-treated parasites to their untreated controls were performed using two-tailed Mann Whitney *U* tests: *p < 0.05 and **p < 0.01. (**e** and **h**) Representative overlay plots of parasite survival (left y-axis) with ‘free’ heme amounts (right y-axis) as a function of (**e**) CQ or (**h**) ZY19489 concentrations. Data were fitted to the sigmoidal concentration response (variable slope) equation in GraphPad Prism.

We assessed the timing of ZY19489’s anti-ABS action by measuring its potency against synchronized rings, trophozoites, and schizonts that had been treated separately for 12 hours^27^. For optimal drug accumulation within the putative site of action, we used the drug-sensitive 3D7 strain that expresses wildtype isoforms of the drug/solute transporters PfCRT and PfMDR1. We included dihydroartemisinin, CQ and the 2-aminopyridine MMV390048 (a phosphatidylinositol 4-kinase inhibitor) as control compounds with established peak activity against early rings, trophozoites and schizonts^27^. These compounds yielded mean IC_50_ values of 1.7 nM, 12.3 nM and 24.4 nM, respectively (Supplementary Table 12). ZY19489 exerted optimal activity against rings and schizonts (mean IC_50_ values of 9 nM and 12.8 nM, respectively, compared to 28.3 nM against trophozoites; Fig. 4a; Supplementary Table 12), a profile distinct from our control compounds.

### ZY19489 inhibits parasite growth by interfering with hemoglobin catabolism

Our observation of swollen DVs in ZY19489-resistant parasites (Fig. 3a) was reminiscent of a similar morphological feature in PPQ-resistant parasites previously associated with defective hemoglobin (Hb) breakdown^28^. To test whether the ZY19489 MoA might also be linked to Hb catabolism, we incubated synchronized 3D7 and Dd2 trophozoites with 10× IC_50_ of ZY19489 for 2.5 hours and compared the ensuing metabolic responses with no-drug controls^29^. Parasites treated with the reference drug ATQ exhibited significant accumulation of *N*-carbamoyl-aspartate and dihydroorotate, two pyrimidine biosynthetic intermediates upstream of the *P. falciparum* dihydroorotate dehydrogenase (PfDHODH; PF3D7_0603300) target (Supplementary Table 13; Supplementary Fig. 6). These changes were accompanied by reduced levels of intracellular uridine monophosphate (UMP), diphosphate (UDP) and triphosphate (UTP), consistent with ATQ-mediated inhibition of the mitochondrial electron transport chain complex and concomitant loss of PfDHODH activity^30^. ZY19489-treated 3D7 and Dd2 parasites, on the other hand, showed significant reductions in short Hb-derived peptides, commonly linked with inhibition of Hb endocytosis and/or catabolism (Fig. 4b; Supplementary Table 13). These short peptides ranged from 2 to 6 amino acids in length, had isoelectric points of four to six, and were negatively charged at pH 5.5 or pH 7.4 (reflecting conditions in the DV lumen or cytosol, respectively) (Supplementary Table 14). We also observed significantly elevated levels of deoxyuridine monophosphate (dUMP), a crucial precursor in the production of thymidine required for DNA replication (Supplementary Table 13; Supplementary Fig. 6). Separately, we explored whether ZY19489 might target cGMP dependent protein kinase (PfPKG; PF3D7_1436600), given this compound’s structural similarity with the aminopyrimidine PfPKG inhibitor ML-10^31^. ZY19489 was found to be inactive against recombinant purified PfPKG (Supplementary Fig. 7).

### ZY19489 inhibits hemoglobin breakdown but not heme detoxification

Hb degradation is initiated by hemoglobinases and leads to generation of amino acids for growth, with ‘free’ heme released as a toxic byproduct. In its labile form, ‘free’ heme can cause oxidative damage to the parasite and is promptly sequestered into inert hemozoin (Hz) in a process that may be accelerated by *P. falciparum* heme detoxification protein (PfHDP; PF3D7_1446800) in complex with hemoglobinases including falcipains^32^. To understand whether ZY19489 inhibits this phase of Hb catabolism, we tested for possible interaction with PfHDP and/or falcipain 2. We generated an aTc-regulatable PfHDP cKD line (designated herein as Dd2^HDP cKD^) and observed no difference in ZY19489 susceptibility at various aTc concentrations compared to control lines (Supplementary Fig. 8). These data argue against any link between ZY19489 anti-ABS activity and inhibition of PfHDP. On the other hand, quinine displayed a significant ~2-fold IC_50_ shift in PfHDP cKD parasites under no aTc relative to parasites cultured at high aTc concentration. We found no significant difference in ZY19489 activity against 3D7 falcipain 2A or 2B knockout lines^33^ compared to the parental 3D7 line (Supplementary Fig. 9). As a control, we included E64d that is a membrane-permeable derivative of the epoxysuccinate inhibitor, E64, whose broad-spectrum antiprotease activity includes falcipain inhibition^33^. E64d exhibited high IC_50_ values against parasites lacking falcipain 2A or 2B (Supplementary Fig. 9).

We then tested for direct interference of Hz formation by first assessing inhibition of the conversion of hematin to β-hematin in a cell-free set-up^34^. ZY19489 failed to block β-hematin formation (IC_50_ >500 μM) in contrast to CQ and amodiaquine that were potent inhibitors with mean IC_50_ values of 19 μM and 6 μM, respectively (Fig. 4c). Secondly, to test whether the effect of ZY19489 on the pathway was only discernible in live parasites, we interrogated a concentration-dependent effect of the drug on three heme species, namely Hb (undigested Hb species), ‘free’ heme (potentially oxidative labile species liberated from Hb proteolysis), and Hz (inert crystalline species) fractioned from mature trophozoites^35^. Inhibitors that complex with heme customarily show a concentration-dependent decrease in Hz levels accompanied by a corresponding increase in ‘free’ heme, which correlates with parasite killing^35^.

In CQ-treated NF54 parasites, the mean ± SEM of ‘free’ heme iron (Fe) present in femtogram per cell (fg/cell) was significantly higher in the highest treatment concentration compared to the untreated control (8.3 ± 0.3 fg/cell *vs* 5.5 ± 0.2 fg/cell; *p* < 0.05; Mann-Whitney *U* tests) (Fig. 4d; Supplementary Table 15). This increase in ‘free’ heme was concentration-dependent and corresponded with a significant decrease in Hz (Fig. 4d; Supplementary Table 15). To interpret the physiological relevance of these observations we superimposed 72-hour parasite growth inhibition curves on the ‘free’ heme levels and observed a correlation between heme concentrations and parasite growth inhibition (Fig. 4e). These data suggest that parasite death was a consequence of the buildup of toxic heme. We also observed modest but significant elevation in Hb levels that plateaued between CQ concentrations corresponding to 1.5× and 2.5× IC_50_ (Fig. 4f; Supplementary Table 15).

In contrast, the levels of ‘free’ heme in ZY19489-treated parasites showed no concentration-dependent pattern (5.6 ± 0.2 fg/cell in untreated control *vs* 6.0 ± 0.1 fg/cell in parasites treated with 2.5× IC_50_) (Fig. 4g). Decreasing amounts of Hz in ZY19489-treated parasites were only observed at higher concentrations and showed no association with ‘free heme levels (Fig. 4g; Supplementary Table 15). Additionally, levels of ‘free’ heme did not correlate with 72-hour parasite survival (Fig. 4h, 4i). Similar lack of inhibition was observed in parasites treated with the antifolate, pyrimethamine (Supplementary Table 15). Nonetheless, treatment with ZY19489 caused significant increases in Hb levels at 1.5× and 2.0× IC_50_ concentrations (Fig. 4i), suggesting some activity of this compound during earlier steps of Hb catabolism.

## Discussion

Based on its potency, safety and pharmacokinetics, ZY19489 has advanced into Phase II clinical trials to treat uncomplicated *P. falciparum* malaria. Its incorporation into non-ACTs is timely given the rapidly expanding list of countries in sub-Saharan Africa where artemisinin partial resistance is present^3^. Here, we uncover key insights into pathways to ZY19489 resistance and its mode of antiparasitic action. *In vitro* drug pressure experiments and WGS revealed a novel PfCRT N246H mutation, whose role in resistance was validated using gene editing. Genetic cross analysis further implicated PfCRT as a primary modulator of susceptibility to ZY19489. Mutant PfCRT N246H parasites had swollen DVs and heightened susceptibility to PPQ and other DV-acting aminoquinolines including the former gold-standard CQ. Mutant parasites demonstrated a substantial fitness cost, with ABS growth rates reduced by 60–75% compared to isogenic parasite lines lacking this mutation. Encouragingly, ZY19489 maintained potency against parasites carrying mutant *pfcrt* and *pfmdr1* alleles present in the field. This compound also did not select for in vivo resistance in combination studies with multiple antimalarials, conducted in *P. falciparum*-infected humanized mice. Using metabolomics and Hb degradation assays, we also observed ZY19489-mediated perturbations of Hb catabolism and detected the accumulation of pyrimidine precursors. Our results offer insight into the potential MoA of ZY19489 and present a genetic marker to screen for potential resistance in the field.

Mutations in *pfcrt* have been shown to modulate the activity of antimalarial drugs in clinical use and under development. The N246H mutation conferred an 11-, 8- and 5-fold increase in mean ZY19489 IC_50_ values against parasites with Dd2, GB4 and FCB *pfcrt* alleles, respectively, highlighting the influence of different PfCRT haplotypes in modulating resistance. By QTL mapping of genetic cross progeny, PfCRT was also associated with decreased susceptibility to PPQ and the structurally distinct compounds MMV665939 and MMV675939^15^, highlighting the influence of this transporter on the activity of diverse antimalarial scaffolds. Of note, the chromosome 7 segment identified in our QTL mapping also included HECT-type E3 ubiquitin ligase UT (PF3D7_0704600), which was previously associated with altered responses to quinine and quinidine and was co-selected with mutant *pfcrt*^36^.

Our molecular dynamics analysis maps PfCRT N246H within the negatively charged central cavity close to the parasite cytosol. CQ and PPQ resistance-conferring mutations localize to specific helices that line this cavity, suggesting this as a primary site of drug interaction^16,37,38^. The N246H mutation faces away from the cavity into the cytosol, potentially causing a conformational change that might affect the transport of PPQ and other 4-aminoquinolines. Importantly, parasites expressing the N246H mutation were sensitized to DV-acting antimalarials including PPQ and CQ, and transport of these drugs by related PfCRT variants was potently inhibited in the presence of ZY19489. If appropriately matched for pharmacokinetics, we envisage that this collateral sensitivity and reciprocal drug-transport phenomenon could be exploited to develop a resistance-refractory combination of ZY19489 + PPQ. In such a scenario, our data suggest that two drugs would theoretically constitute an effective combination that would force *pfcrt* into an “evolutionary trap” and prevent the emergence of high-grade resistance to either. This concept has been demonstrated for other antimalarials, including PfDHODH, Pf20S proteasome and mitochondrial bc1 complex inhibitors^10^, and is one of the rationales behind current triple ACT therapies. For instance, amodiaquine paired with artemether + lumefantrine or mefloquine partnered with dihydroartemisinin + PPQ, exert reciprocal selective pressures associated with opposite shifts in the prevalence of mutant versus wild-type isoforms of PfCRT and PfMDR1^39–41^. To further consolidate this line of argument, our results dispel concerns of cross resistance between ZY19489 and PPQ or other antimalarials whose clinical efficacy can be compromised by currently circulating mutant *pfcrt* and *pfmdr1* alleles. This is instructive to the ongoing Phase II trials in Gabon where mutant *pfcrt*, which could provide a potential background for N246H to emerge, constituted an estimated 53% of all samples in a recent report^42^. Nonetheless, the high resistance barrier of ZY19489 and steep fitness cost associated with PfCRT N246H suggest that clinical resistance to this antimalarial is unlikely to arise as mutant parasites would likely be rapidly outcompeted in the field.

The significant attenuation of short Hb-derived peptide levels in ZY19489-treated parasites suggests that perturbation of Hb endocytosis and/or catabolism constitutes one mechanism through which this compound exerts its antiplasmodial activity. This metabolic fingerprint corroborates a prior clustering of ZY19489 with CQ^29^. Further, aberrant accumulation of peptides has been observed in variant PfCRT isoforms where it is associated with reduced parasite fitness, presumably from deprivation of essential globin-derived amino acids^28,43,44^. Our analysis identified HVDD (a truncated version of HVDDM) and VDPVNF, both of which were demonstrated to be putative PfCRT substrates in [^3^H]-CQ *cis*-inhibition experiments in *Xenopus laevis* oocytes^45^. The absence of direct interaction between ZY19489 and ‘free’ heme, or the putative Hz nucleation factor PfHDP, or the hemoglobinases falcipains 2A and 2B, suggests inhibition of other targets within the Hb catabolism pathway. Our metabolomic analysis also revealed an accumulation of deoxyuridine monophosphate, which could imply a potential impact on pyrimidine biosynthesis. This likelihood of an additional MoA could help explain the compound’s high barrier for resistance. Moreover, Hb uptake and degradation begins in early rings^46^ and pyrimidine biosynthesis peaks during the trophozoite to schizont transition^47^, which would align with our observations of stage-specific potency against rings and schizonts.

In summary, we have provided evidence of a novel PfCRT mutation that mediates low-level resistance to ZY19489. Importantly, the resultant mutants have significant growth defects and are sensitized to DV-acting 4-aminoquinolines. Inhibition of mutant PfCRT-mediated PPQ transport by ZY19489 opens the exciting possibility of partnering these two drugs as a way to restrict *pfcrt* from mutating to confer multidrug resistance, potentially producing an effective resistance-refractory non-ACT combination.

## Methods

### Ethics Statements

The studies performed at The Art of Discovery were approved by The Art of Discovery Institutional Animal Care and Use Committee, which is certified by the Biscay County Government (Bizkaiko Foru Aldundia, Basque Country, Spain) to evaluate animal research projects from Spanish institutions according to point 43.3 from Royal Decree 53/2013, from 1st February 2013 (BOE-A-2013–1337). All experiments were carried out in accordance with the European Directive 2010/63/EU. The results from the animal experiments are reported following ARRIVE guidelines except for disclosure of business trade confidential information (https://www.nc3rs.org.uk/arrive-guidelines). The human biological samples were sourced ethically, and their research use was with informed consents.

### Generation of *P. falciparum* V-type ATPase subunit D conditional knockdown parasite lines

This cKD line was generated by fusing the coding sequence and non-coding RNA aptamer sequences in the 5’ and 3’ untranslated regions (UTRs), permitting translational regulation using the TetR-DOZI system^48,49^. Gene editing was achieved by CRISPR/*Sp*Cas9 using the linear pSN054 vector that contains cloning sites for the left homology region (LHR) and the right homology region (RHR) as well a gene-specific guide RNA under control of the *T7* promoter. Cloning into the pSN054 donor vector was carried out as described previously^48,49^. The vector includes preinstalled V5–2×HA epitope tags, a 10× tandem array of TetR aptamers upstream of an *Hsp86* 3’UTR, and a multicistronic cassette for expression of TetR-DOZI (regulation), blasticidin S-deaminase (the selection marker) and a Renilla luciferase (*RLuc*) reporter. The LHR and recoded region were installed in-frame with tandem V5–2×-hemagglutinin (HA) tags to generate a C-terminal epitope-tagged V1-A coding sequence, upstream of the regulatory aptamer array. The final construct was sequence-verified and further confirmed by restriction digests. Transfection into Cas9- and T7 RNA polymerase-expressing NF54 parasites was carried out by pre-loading erythrocytes with the donor vector^50^. Parasite culture was maintained continuously in 500 nM anhydrotetracycline (aTc, Sigma-Aldrich 37919). Drug selection with 2.5 μg/mL of Blasticidin S (RPI Corp B12150–0.1) was initiated four days after transfection. Cultures were monitored by Giemsa smears and Renilla luciferase (RLuc) measurements.

### Verification of conditional knockdown cKD strategy

To assess regulation of the PfV1-D protein expression, cKD parasites were cultured in the presence (500 nM) and absence of aTc. Protein samples were extracted after 72 hours via saponin lysis and resuspended in parasite lysis buffer that consists of 4% SDS and 0.5% Triton X-114 in PBS. Proteins were separated on a Mini-PROTEAN^®^ TGX^™^ Precast Gels (4–15% gradient) in Tris-glycine buffer, transferred to a polyvinylidene fluoride (PVDF) membrane using the Mini Trans-Blot Electrophoretic Transfer Cell system according to the manufacturer’s instructions, and blocked with 100 mg/mL skim milk in TBS/Tween. Membrane-bound proteins were probed with mouse anti-HA (1:3000; Sigma H3663) and rabbit anti-GAPDH (1:5000; Abcam AB9485) primary antibodies, and anti-mouse (1:5000; Thermo Fisher Scientific 62–6520) and anti-rabbit (1:5000; Cell signaling 7074S) horseradish peroxidase (HRP)-conjugated secondary antibodies. Following incubation in SuperSignal^®^ West Pico Chemiluminescent substrate (Thermo Fisher Scientific PI34080), protein blots were imaged and analyzed using the ChemiDoc^™^ MP System and Image Lab 5.2.0 (Bio-Rad).

### Assessment of parasite growth and compound susceptibility assays using cKD lines

Assessment of parasite proliferation rate upon protein perturbations was carried out using luminescence as a readout of growth. Synchronous ring-stage parasites cultured in the presence (50 nM) or absence of aTc were set up in triplicate wells in 96-well U-bottom plates (Corning^®^ 62406–121). Luminescence signals were quantified at 0 and 72 hours after incubation using the Renilla-Glo(R) Luciferase Assay System (Promega E2750) and the GloMax^®^ Discover Multimode Microplate Reader (Promega). The luminescence values in the knockdown condition (6 nM aTc) were normalized to 50 nM aTc-treated (100% growth) and 200 nM dihydroartemisinin-treated (no growth) samples. Results were visualized on bar graphs using GraphPad Prism (version 10).

To assess compound activity, ZY19489 and bafilomycin A (a V-type ATPase inhibitor) were serially diluted in complete tissue culture medium. Synchronous ring-stage V1-D cKD and control parasites were maintained in high (50 nM) and low (6 nM) aTc or no aTc (for the control line) and were distributed into the drug plate. DMSO and 200 nM dihydroartemisinin treatments served as reference controls. Luminescence was measured after 72 hours as described above and IC_50_ values were obtained from dose-response curves using GraphPad Prism.

### *P. falciparum* resistance selections with ZY19489

*In vitro* selection was conducted using a pulsing (intermittent drug pressure) method on Dd2-Polδ. This is a Dd2 parasite line with defective proof-reading activity following CRISPR/Cas9-based replacement of D308 and E310 - the two conserved catalytic residues of *P. falciparum* DNA polymerase δ, with alanine. This impaired proof-reading activity endows Dd2-Polδ with a ~5-fold higher mutability rate in the presence of a drug compared to Dd2^12^. Briefly, 10^9^ parasites in triplicate flasks were exposed to 10× ZY19489 (100 nM) for 11 days until all parasites cleared. The cultures were thereafter passaged in drug-free media with weekly introduction of fresh red blood cells (RBCs) until day 34 when parasites reappeared in two flasks. *In vitro* drug sensitivity testing confirmed these recrudescent parasites were still susceptible to ZY19489 and subsequently pressured again with 100 nM ZY19489 for another 16 days. During this period, parasites in the third flask also recrudesced but were also exposed to drug since there was no shift in their drug response. Upon clearance on day 50, cultures were again passaged in drug-free media until day 71 when they reappeared. All flasks still had drug sensitive Dd2-Polδ parasites and were exposed to another round of 100 nM ZY19489 for 19 days until all parasites cleared. Drug pressure was then removed until day 112 when only one flask recrudesced. We further pressured the parasites from this flask for another 12 days before removing drug. On day 136, recrudescent parasites appeared in this flask, which upon testing showed a decrease in drug sensitivity compared to untreated Dd2-Polδ parasites. No parasites were recovered from the other two flasks after day 160 and those selections were halted. Clones were obtained from the resistant bulk cultures by limiting dilution. IC_50_ values were determined in 72-hour drug assays, with parasitemia measured by flow cytometry of cultures stained with SYBR Green and MitoTracker Deep Red (Life Technologies), with ~10,000 cells analyzed per well using an iQue Plus (Sartorius).

### 72-hour *in vitro* drug susceptibility assay

Susceptibility to all drugs tested in this study was measured by incubating asynchronous, ABS parasites plated at 0.2% to 0.3% parasitemia (or 0.8% for the poorly growing ZY19489-resistant lines harboring the *pfcrt* N246H allele) and 1% hematocrit at 37° C in 96-well plates. The assays were set up across a range of drug concentrations with two-fold dilutions. For the experiments testing for susceptibility of recrudescent parasite lines that emerged in humanized NSG mice to ZY19489, M5717, MMV693183, or ferroquine, parasites were cultured at 2% hematocrit in human O^+^ or A^+^ RBCs in RPMI-1640 media, supplemented with 25 mM HEPES (Fisher), 50 mg/L hypoxanthine (Sigma Aldrich), 2 mM L-glutamine (Cambridge Isotope Laboratories, Inc.), 0.21% sodium bicarbonate (Sigma Aldrich), 0.5% (w/v) Albumax (Invitrogen), 8% filtered, heat-inactivated, pooled off-the-clot AB^+^ human serum (Interstate Blood Bank, Inc.), and 10 μg/mL gentamicin (Fisher). The parental *P. falciparum* Pf3D7^0087/N9^ strain that has been adapted to proliferate in this humanized NSG mouse model was cultured in parallel as the drug-sensitive parental control strain. All compound susceptibility assays for the recrudescent lines and parental control strain were performed using serum-containing media. Parasite growth in each well was assessed after 72 hours using flow cytometry of cultures stained with SYBR Green I and MitoTracker Deep Red. IC_50_ and IC_90_ values were determined by nonlinear regression analysis (GraphPad Prism 7). Unless otherwise stated, assays were repeated on four independent occasions with technical duplicates. Statistical comparisons were made using Mann–Whitney *U* tests.

### Whole-genome sequence analysis

DNA from infected RBCs was extracted using the QiAmp DNA Blood Mini kit (Qiagen). The samples were then pooled and sequenced on an Illumina MiSeq flow cell to obtain 300 bp paired end reads. The sequence data were aligned to the Pf3D7 reference genome (PlasmoDB 48_Pfalciparum3D7; https://plasmodb.org/plasmo/app/downloads/release-48/Pfalciparum3D7/fasta/) using the Burrows-Wheeler Alignment (BWA version 0.7.17). PCR duplicates and unmapped reads were filtered using Samtools (version 1.13) and Picard MarkDuplicates (GATK version 4.2.2). The reads were realigned around indels using GATK RealignerTargetCreator and base quality scores were recalibrated using GATK Table-Recalibration. GATK HaplotypeCaller (version 3.8) was used to identify all possible variants in clones which were filtered based on quality scores (variant quality as function of depth QD > 1.5, mapping quality > 40) and read depth (depth of read > 5) to obtain high-quality single nucleotide polymorphisms that were annotated using snpEFF. The list of variants from resistant clones were compared against the Dd2-Polδ parental clone to obtain homozygous single nucleotide polymorphisms present exclusively in the resistant clones. Integrated Genome Viewer was used to confirm polymorphisms present in resistant clones. BIC-Seq version 1.1.2 was used to discover copy number variants against the parental strain using the Bayesian statistical model.

### Targeted sequencing of *PfeEF2* and analysis of mutations

The gene encoding *Pf*eEF2 (PF3D7_1451100), which mediates resistance to M5717, was PCR-amplified and sequenced as previously described^26^. Sequences were aligned to wildtype *PfeEF2* from the 3D7 genome reference strain and analyzed using Geneious 9.1.8. Electropherograms were visually inspected to identify mixed sequences indicating multiple subpopulations.

### Gene editing using zinc-finger nucleases and CRISPR/Cas9 strategies

*pfcrt* was edited using a customized two-plasmid zinc-finger nuclease approach that replaces the endogenous allele with a recombinant allele containing the mutations of interest^13^. Mutations were introduced into *pfcrt*^Dd2^, *pfcrt*^GB4^ and *pfcrt*^FCB^ donor plasmids by site-directed mutagenesis. The D233N mutation was inserted through CRISPR/Cas9 editing, using the previously described “all-in-one” method^51^, with a 610 bp repair template. Two gRNAs were selected based on their selectivity and their proximity to D233, and gRNA primers were annealed and cloned into the pDC2 vector at the BbsI restriction site as previously described^51^. Repair templates containing the D233N mutation and silent shield mutations were synthesized and cloned into the pDC2 vector by In-Fusion cloning at the EcoRI/AatII sites. Dd2 *P. falciparum* ABS parasites were cultured in RBCs in RPMI 1640-based culture media containing 0.5% w/v Albumax. RBCs were purchased from the Interstate Blood Bank (Memphis, TN) as pooled, de-identified, anonymized blood that was washed to remove any residual leukocytes. Approval for this protocol (AAAU3761) was provided on 28 October 2022 by the Columbia University Institutional Review Board, which deemed this work to be Not Human Subjects Research Under 45 CFR 46. Parasites were cultured at 37° C in an airtight chamber with 5% O_2_/ 5% CO_2_/ 90% N_2_. Ring-stage parasites were electroporated with 50–100 μg of purified plasmid DNA in Cytomix. The donor plasmids carrying the human *dhfr* marker were selected with 2.5 nM WR99210 (Jacobus Pharmaceuticals), and the zinc-finger nuclease plasmid harboring blasticidin S-deaminase was selected with a 6-day pulse of 2 μg/ml blastidicin hydrochloride. Editing was confirmed using PCR and Sanger sequencing, and clones were obtained by limiting dilution.

### Quantitative trait locus (QTL) analysis and mapping

The generation of the genetic cross between NF54 and RF7, cloning and whole-genome sequencing of the progeny were conducted as described previously^15^. Unique recombinants were identified using whole-genome sequencing. The R package (version 2) was used to map QTL peaks. To identify significant QTLs for each drug response phenotype, 1,000 permutations of phenotypic data (IC_50_) were performed to obtain a distribution of maximum log of the odds (LOD) scores. These scores were then used to calculate the LOD threshold at 95% probability. Fine mapping of the QTL segments was performed using Bayesian interval mapping at a 95% confidence level.

### Modeling PfCRT in the open-to-DV and open-to-cytosol conformations

The cryo-EM structure of PfCRT strain 7G8 (PDB: 6UKJ)^16^ was used as the wildtype protein template. To include the missing residues (114–122), we employed AlphaFold2, with the original PDB structure serving as the template for modeling. The same approach was used for the N246H mutant, substituting asparagine at position 246 for histidine in the primary sequence. The resulting wildtype and N246H structures were highly similar, with a root-mean-square deviation (RMSD) of less than 1 Å across all residues. The open-to-cytosol conformation of PfCRT was obtained from previous work^16^. The missing segments and residues (81–88, 110–125, 148–154, 169–180, 198–214, 235–251, 269–322, 336–344, 364–381) were modeled using SwissModel^52^, except for the 277–315 region, which was truncated due to the presence of two disulfide bridges. The final model exhibited an RMSD of 0.9 Å, calculated over 143 pruned atom pairs, when compared to the open-to-cytosol structure from Berger and colleagues^53^, indicating a high degree of structural similarity between the two models. The same procedure was employed to model the mutated N246H.

### Molecular dynamics simulation

The protonation states of the amino acids in all models were assigned using PDB2PQR^54^, with pH values of 5.5 for the open-to-DV conformation and 7.0 for the open-to-cytosol conformation. The ionization state of ZY19489 was checked using MolGpKa^55^. To obtain an initial pose of the ligand in each system, molecular docking was performed using GOLD, defining a 12 Å radius around the alpha carbon of residue 246 as the binding site. The initial 3D structure of the ligand was generated with Omega^56^. The 7G8 and 7G8+N246H docking poses obtained for the open-to-DV and open-to-cytosol systems were then embedded into an explicit lipid bilayer using CHARMM-GUI Membrane Builder^57^. The membrane composition included 1-palmitoyl-2-oleoyl-sn-glycerol-3-phosphocholine (POPC), 1-stearoyl-2-oleoyl-sn-glycero-3-phosphoethanolamine (SOPE) and cholesterol (CHL) in a 4:3:3 ratio, based on the approach from Berger and colleagues^53^. A 150 mM NaCl concentration was added to neutralize the system. The systems were solvated with TIP3P (transferable intermolecular potential with 3 points) water molecules, resulting in approximately 131k atoms per system.

For the force fields, ff14SB was used for the protein, Lipid21 for the lipids, and GAFF2 for the ligand, with atomic charges derived using AM1-BCC. All-atom molecular dynamics simulations were performed in Amber24 using pmemd.cuda. The systems were initially energy-minimized using the steepest descent method for 10,000 steps. For equilibration, we followed the six-step protocol from CharmmGUI, which includes two NVT simulations with the Langevin thermostat and four NPT simulations at 303.15 K. Finally, a 300 ns production run was performed in triplicate for each system, totaling 3.6 μs of simulation time. To analyze the results from the molecular dynamics simulations, we used cpptraj and MDAnalysis^58^. Representative conformations of each system were obtained through k-means clustering, generating 10 clusters over 500 iterations, based on the ligand-protein RMSD for heavy atoms only.

### Drug transport assays

PfCRT 7G8 and 7G8+F145I variants were purified and inhibition of CQ and PPQ uptake was measured using PfCRT-containing proteoliposomes as described previously^16^ with the following modifications. Purified PfCRT variants were reconstituted in preformed liposomes made of *Escherichia coli* total lipids:cholesteryl hemisuccinate in a ratio of 97:3 (w/w) and the lumen of the proteoliposomes was composed of 100 mM KPi, pH 7.5 and 2 mM β-mercaptoethanol. Uptake of 100 nM [^3^H]CQ (1 Ci/mmol) or 100 nM [^3^H]PPQ (1 Ci/mmol) was performed by diluting PfCRT-containing proteoliposomes (30 ng of PfCRT per reaction) in an uptake buffer containing 100 mM Tris/MES, pH 5.5, in the presence or absence of 1 μM of the test compounds. In addition, 1 μM valinomycin was added to the reaction to generate a K^+^ diffusion potential-driven membrane potential (ΔΨ). Reactions were stopped after 30 seconds by the addition of ice-cold 100 mM KPi, pH 6.0, and 100 mM LiCl, and filtered through 0.45 μm nitrocellulose filters (Millipore). Filters were dried and incubated in a scintillation cocktail, and the radioactivity captured on the filters was counted in a Hidex SL300 scintillation counter. The efficiency of detection was calculated with a standard curve of known concentrations of each radiolabeled compound, and this was used to transform decays per minute (dpm) into pmol. The nonspecific interaction of each compound with nitrocellulose filters was determined by measuring mock uptake in the absence of liposomes or proteoliposomes. These values (determined for each experiment) were used to calculate background uptake in liposomes or proteoliposomes. Drug-specific uptake was determined by subtracting the time-dependent accumulation of the tested compounds in control liposomes (lacking PfCRT) from that measured in PfCRT-containing proteoliposomes^16^.

### *In vitro* growth rate and fitness assay

We measured relative growth rates of the *pfcrt* mutant parasite lines by tracking the growth of the parasites and their isogenic parents over eight generations. Briefly, all parasites were seeded at 0.2% starting parasitemia and 2% hematocrit on day 0 with fresh RBCs. The parasites were then allowed to grow undisturbed for the first 3 days, after which parasitemia was assessed by flow cytometry. Media was changed without disturbing the settled culture and culturing was continued until day 4 when parasitemia was measured again. The parasite cultures were cut back to 0.2% parasitemia on day 4, with the dilution ratio recorded. Parasitemia measurements were subsequently done on day 6, 8, 10, 12, 14 and 16, with dilutions made back to 0.2% parasitemia for any cultures that had expanded to >2% parasitemia. These experiments were conducted on three different occasions with technical duplicates. Parasite growth rate was computed as the square root of parasitemia on the day of dilution divided by 0.2% (starting parasitemia) for days 4, 8, 12 and 16. Mean growth rate was obtained from the average of this analysis over three biological repeats. The mean growth rate for the mutant lines was then computed as a proportion of the isogenic parent. The mean fitness cost associated with PfCRT N246H mutation was calculated relative to its isogenic parent using the following formula:

$$\%\;Mean\;{FC\;}^{pfcrtN246H}\;=\;100\times (1-\;({MGR\;}^{mutant}\;÷\;{MGR\;}^{parent}))$$

where ${FC\;}^{pfcrtN246H}$ is the fitness cost associated with the N246H mutant relative to the parental line and MGR is the mean growth rate per two-day cycle.

### Stage specific profiling of antimalarial activity

Determination of the stage-specific activity of ZY19489 and control compounds was conducted as reported elsewhere with minor modifications^27^. Briefly, mature schizonts were obtained via Percoll gradient purification. Upon reinvasion of RBCs, cultures were synchronized by sorbitol treatment to obtain pure early rings. The 40 – 42 hours ABS cycle duration of 3D7 was exploited to allow for three different parasite incubation phases corresponding to rings (0 – 12 hours), trophozoites (18 – 30 hours) and schizonts (30 – 42 hours). Culture synchronicity was confirmed by microscopic observation. After each incubation period (12 hours), drugs were removed from the culture through three rounds of washing using pre-warmed media followed by plate change. All pipetting and washing steps were performed using a TECAN Freedom Evo 100 for increased throughput, accuracy and to avoid cross-contamination of wells. Each set of plates per timepoint was placed in a separate humidified chamber to avoid any delay in growth rate due to temperature variations. For the three different stages, growth inhibition was assessed at the 60-hour time point, at which time parasites had expanded, reinvaded new RBCs, and developed into trophozoites to allow for accurate quantification by flow cytometry. Parasite survival for both the 72 hours and stage-specific 12-hour exposures was assessed by SYBR Green and MitoTracker Deep Red FM staining (Life Technologies) and subsequent flow-cytometric analysis (IntelliCyt iQue3, Sartorius). IC_50_ values were derived from growth inhibition data using nonlinear regression (Prism 7, GraphPad) following six independent biological repeats with two technical replicates.

### Sample preparation for untargeted LC-MS metabolomics

3D7 and Dd2 *P. falciparum* parasite strains were cultured under standard conditions at 2% hematocrit and tested for *Mycoplasma* using a MycoAlert PLUS Mycoplasma Detection Kit (Lonza). Excluding the cycle preceding sample collection, the *Mycoplasma*-free parasites were sorbitol-synchronized in each generation for at least two generations and subsequently cultivated to the trophozoite stage with a parasitemia of ~10%. Infected RBCs were then magnetically purified using MACS CS columns and VarioMacs magnets. Metabolite extracts were obtained from the purified infected RBCs treated for 2.5 hours with concentrations corresponding to 10× IC_50_ of the atovaquone or ZY19489, resulting in a 1 mL extraction composed of 90% MeOH. Extracts were spiked with a 0.5 μM [^13^C_4_,^15^N_1_]-labeled aspartate standard^29^, vortexed, and centrifuged for 10 minutes at 4°C at 15,000 rpm. The supernatant was then transferred into 1.5 mL tubes, dried under a stream of nitrogen, and stored at −80°C.

### Metabolomic profiling of drug-treated parasites via mass spectrometry

Upon thawing on ice, extracts were resuspended in ice-cold HPLC-grade water containing 1μM chlorpropamide to reach a concentration of 10^6^ cells/μL. After vortexing and centrifugation for 10 minutes at 4°C at 15,000 rpm, supernatants were transferred into 800 μL CRIMP vials in preparation for mass spectrometry. Samples were fractionated using an XSelect HSS T3 2.5 μm C18 Waters column with a 25-minute gradient of 3% aqueous methanol, 15 mM acetic acid, and 10 mM tributylamine and were processed in negative ionization mode on a Thermo Exactive Plus orbitrap.

Data acquired from the mass spectrometry were imported into the el-MAVEN software package for peak picking^59^ and were standardized using the [^13^C_4_,^15^N_1_] aspartate standard. Fold changes were computed and uploaded onto Rstudio (http://www.rstudio.com/) employing the Hyperspec and Suprahex packages^60^. Metabolic profiles, as represented by log_2_ fold changes, were transferred onto a hexagonal map featuring 113 metabolites.

### Inhibition of recombinant *P. falciparum* cGMP-dependent protein kinase (PfPKG)

Full length PfPKG (PF3D7_1436600) was expressed in *OverExpress*^™^ C41(DE3) pLysS Chemically Competent Cells (Sigma Aldrich, CMC0018) using an adapted methodology described previously^31,61^. Briefly, the N-terminal His-tagged recombinant PfPKG protein was purified using a TALON column (GE Healthcare). The final buffer composition of purified protein was 25 mM HEPES pH 7.5, 20 mM NaCl, 120 mM KCl, and 5% glycerol. Purified protein was concentrated using a 15-mL Amicon Ultra 50 KDa MWCO concentrator (Merck Millipore, UFC905024). PfPKG IC_50_ assays were performed based on previously described methods using the ADP-Glo Kinase Assay (Promega) to measure ADP formation^62^. Briefly, 3-fold serial dilutions of each inhibitor were prepared in 100% DMSO, and inhibitors were subsequently diluted into assay buffer (25 mM HEPES pH 7.4, 0.1 mg/mL BSA, 0.01% (v/v) Triton-X 100, 20 mM MgCl_2_, 2 mM DTT, 10 μM cGMP) to 10× the final required concentration. 0.5 μL of each inhibitor dilution was transferred into a white 384-well plate (Greiner #781075). 4.5 μL of a mix containing ATP, GRTGRRNSI-NH2 and PfPKG in assay buffer was then added to each well. The final 5 μL kinase reaction contained 3 nM PfPKG protein, 10 μM ATP, 20 μM GRTGRRNSI-NH2, 1% (v/v) DMSO and inhibitor in assay buffer. Reactions were incubated for 30 minutes at 22°C.

ADP formation was measured using the ADP-Glo Kinase Kit (Promega). Briefly, 5 μL ADP-Glo reagent was added to each well and incubated for 30 minutes at 22°C to deplete the remaining ATP. 10 μL of Kinase Detection Reagent was then added and the reaction incubated for a further 30 minutes at 22°C. The plate was sealed with an adhesive foil seal for all incubation steps. Luminescent signal was measured using a Spectromax Plate Reader. Data were normalized based on the 100% activity controls (1% DMSO only) and the 100% inhibition controls (10 μM kinase inhibitor Staurosporine). Mean IC_50_ values were calculated from N, n = 3,4 independent experiments.

### Generation and drug susceptibility testing of *P. falciparum* heme detoxification protein cKD lines

PfHDP cKD parasites were generated using a CRISPR/Cas9-based TetR-DOZI aptamer system^49^. Briefly, the left homology region fused with recodonized 3’ end of PfHDP and right homology region as well as guide sequence were cloned into a pSN054 donor plasmid containing a 3×HA epitope tag at the 3’ end of the coding sequence. The final construct together with the pDC2-cam-coSpCas9-U6-gRNA-hDHFR plasmid carrying the guide sequence was transfected into Dd2 parasites and maintained in 500 nM aTC and 2.5 μg/mL of blasticidin hydrochloride. Edited parasites were confirmed by site-specific integration PCR.

*In vitro* drug sensitivities of ABS parasites were determined as described above with minor modifications. Asynchronous Dd2 and PfHDP cKD parasite lines were cultured in the presence of 500 nM aTC and then washed to remove aTc. Asynchronous ABS parasites were plated at 0.3% parasitemia and 1% hematocrit in 96-well plates and were incubated with a ten-point, three-fold range of drug concentrations in duplicates with either 30 nM, 3 nM and 0 nM aTc. Plates were incubated at 37°C for 72 hours and parasite survival was assessed by flow cytometry using SYBR Green (Invitrogen) and MitoTracker Deep Red FM (Life Technologies) as nuclear stain and vital dyes, respectively. IC_50_ and IC_90_ values were derived from growth inhibition data using nonlinear regression or linear interpolation (Prism 10, GraphPad) as means ± SEM from four independent biological repeats with two technical replicates.

### Inhibition of *P. falciparum* falcipain 2A and 2B knockout parasites

Falcipain knockout lines were generated as described previously^33^. Susceptibility to ZY19489 and E64d was measured against the 3D7-A10 and 3D7 FP2A/B KO lines by incubating asynchronous, ABS parasites plated at 0.3% (or 0.5% for 3D7 FP2A/B KO) parasitemia and 1% hematocrit at 37°C in 96-well plates. The assays were set up across a range of drug concentrations with two-fold dilutions. Parasite growth in each well was assessed after 72 hours using flow cytometric analysis of cultures stained with SYBR Green I and MitoTracker Deep Red. IC_50_ and IC_90_ values were determined by nonlinear regression analysis. Unless otherwise stated, assays were repeated on four independent occasions in technical duplicates and statistical comparisons were made using Mann-Whitney *U* tests.

### *In vitro* cell-free β-hematin inhibition assay

Inhibition of lipid-mediated hemozoin formation was quantified by measuring the formation of β-hematin dimers from hemin chloride, in a detergent-mediated assay that substitutes neutral lipids for the commercially available lipophilic detergent Nonidet P-40^34^. Unreacted hematin was detected through the formation of *bis*-pyridyl-Fe(III)PPIX complex which absorbs at a wavelength of 405 nm.

Compound stocks (20 mM) of ZY19489, amodiaquine and PYR were prepared in DMSO, while CQ was prepared in water. These stocks were then diluted to 2 mM with a water/NP40 detergent solution, resulting in a final solution of compound in 61.1 mM NP40/10% DMSO. Serial dilutions into this same buffer were then made for each compound. A 25 mM hemin stock solution was prepared by sonicating hemin in DMSO for one minute and then suspending 179 μL of this stock in a 1 M acetate buffer (20 mL, pH 4.8). The homogenous suspension was then added to the wells to give final buffer and hemin concentrations of 0.5 M and 100 mM, respectively. Plates were covered and incubated at 37°C for 5 hours to enable β-hematin formation. A solution of 50% (v/v) pyridine, 30% (v/v) H_2_O, 20% (v/v) acetone and 2 M HEPES buffer (pH 7.4) was prepared and 32 μL added to each well to give a final pyridine concentration of 5% (v/v). Acetone (60 μL) was then added to each well to assist with β-hematin dispersion. The UV-vis absorbance of the plate wells was read on a SpectraMax P340 plate reader. Sigmoidal concentration-response curves were fitted to the absorbance data using GraphPad Prism to obtain an IC_50_ value for each compound.

### Cellular heme fractionation assay

The IC_50_ values used here for ZY19489, CQ and PYR were obtained from drug susceptibility experiments using the 72-hour SYBR Green assay described above. The heme profiles of NF54 parasites treated separately with the three antimalarials were determined as described previously^35^. Briefly, NF54 cultures were synchronized at 48-hour intervals with 5% (wt/vol) sorbitol, and ring-stage parasites at 5% parasitemia and 2% hematocrit were incubated with drug concentrations corresponding to 0.5×, 1×, 1.5×, 2× and 2.5× their IC_50_ values. Untreated infected RBCs were included as control. Parasites were harvested 30 hours post incubation, and the mature trophozoites were isolated with 0.05% (wt/vol) saponin and washed with 1× PBS (pH 7.5) to eliminate traces of residual RBC-derived Hb. The number of trophozoites in these samples were quantified using flow cytometry. The contents of the trophozoite pellet were then released by hypotonic lysis and sonication. Following centrifugation, the supernatants (first fraction) corresponding to membrane-soluble Hb were treated with 4% (wt/vol) SDS and 2.5% (vol/vol) pyridine. Pellets were again treated with 4% SDS, 2.5% pyridine, sonicated, and centrifuged. Supernatants corresponding to the ‘free’ heme fraction (second fraction) were then recovered. The remaining hemozoin pellets (third fraction) were then solubilized in 4% SDS and 0.3 M NaOH, neutralized with 0.3 M HCl, sonicated, and treated with 25% pyridine. The UV-visible spectrum of each heme fraction as a Fe(III) heme-pyridine complex was measured using a multiwell plate reader (Spectramax 340PC; Molecular Devices). The total amount of each heme species was quantified using a heme standard curve^35^. The mass of each heme Fe species per trophozoite was calculated by dividing the total amount of each heme species by the corresponding number of parasites in that fraction^35^. Statistical comparisons were made using two-tailed Mann-Whitney *U* tests using GraphPad Prism.

## Supplementary Material

Supplementary Figures 1 – 9 and Supplementary Tables 1 – 14.

Supplementary Files

This is a list of supplementary files associated with this preprint. Click to download.

• OkomboZY19489SupplementaryMaterial.docx

## Figures and Tables

**Table 1.** Mutations identified from whole-genome sequencing of ZY91489-selected Dd2-Polδ clones.

Gene ID	PlasmoDB annotation	Amino acid change	Clones with mutation

PF3D7_0709000	*P. falciparum* chloroquine resistance transporter	N246H	C8, E3, F7, G2
PF3D7_1217600	anaphase-promoting complex subunit 10, putative	D233N	C8, E3, F7, G2
PF3D7_1321300	conserved *Plasmodium* membrane protein, unknown function	T1776I	C8
PF3D7_1320800	dihydrolipoyllysine-residue succinyltransferase component of 2-oxoglutarate dehydrogenase complex	K242T	C8

**Table 2.** Phenotypic and genotypic profiles of recrudescent *P. falciparum* 3D7^0087/N9^ parasites from humanized NSG mice treated with ZY19489, MMV693183, M5717, or ZY19489-based combination therapies.

Parasite Line ID^a^	NSG mouse treatment group and dosing regimen^a^	IC_50_ ± SEM (nM)^b^		Genetic changes^c^

	**ZY19489+MMV693183**	**ZY19489**	**MMV693183**	
TAD 022	None (control)	11.1 ± 0.6	2.6 ± 0.3	Parental reference
TAD 746	1 × 40 mg/kg ZY19489 + 1 × 1.5 mg/kg MMV693183	11.1 ± 0.5	2.3 ± 0.2	None
TAD 752	1 × 20 mg/kg ZY19489 + 1 × 5.0 mg/kg MMV693183	11.2 ± 0.7	2.6 ± 0.2	None
TAD 755	1 × 20 mg/kg ZY19489 + 1 × 5.0 mg/kg MMV693183	12.3 ± 0.8	2.9 ± 0.1	None
TAD 758	1 × 40 mg/kg ZY19489 + 1 × 1.5 mg/kg MMV693183	11.2 ± 0.2	2.8 ± 0.2	None
TAD 761	1 × 40 mg/kg ZY19489 + 1 × 5.0 mg/kg MMV693183	11.8 ± 0.8	2.8 ± 0.3	None
TAD 764	2 × 40 mg/kg ZY19489 + 1 × 1.5 mg/kg MMV693183	11.7 ± 0.3	2.6 ± 0.3	None
TAD 767	1 × 5.0 mg/kg MMV693183 + 1 × 40mg/kg ZY19489	11.9 ± 0.9	2.6 ± 0.2	None
TAD 770	3 × 2.5 mg/kg MMV693183	11.2 ± 0.5	2.6 ± 0.2	None
TAD 773	1 × 20 mg/kg ZY19489 + 3 × 2.5 mg/kg MMV693183	11.6 ± 0.8	2.9 ± 0.4	None
TAD 779	2 × 40 mg/kg ZY19489 + 3 × 2.5 mg/kg MMV693183	11.0 ± 0.3	2.5 ± 0.3	**Y1020H** in PF3D7_0825000, **N636Y** in PF3D7_1416100
TAD 782	2 × 40 mg/kg ZY19489 + 3 × 2.5 mg/kg MMV693183	11.7 ± 1.2	2.9 ± 0.2	None
	**ZY19489+Ferroquine**	**ZY19489**	**Ferroquine**	
TAD 022	None (control)	6.7 ± 0.6	10.3 ± 1.6	Parental reference
TAD 494	4 × 40 mg/kg ZY19489	7.2 ± 0.5	8.3 ± 0.2	None
TAD 496	4 × 40 mg/kg ZY19489 + 1 × 15 mg/kg Ferroquine	6.9 ± 1.2	9.5 ± 1.1	**D1698N** in PF3D7_0210200, PF3D7_1224000 (loss of 3.5 copies)
TAD 497	4 × 40 mg/kg ZY19489 + 1 × 15 mg/kg Ferroquine	7.1 ± 0.9	8.9 ± 0.6	**N1580S** in PF3D7_1329100, **G48A** in PF3D7_1408600
	**ZY19489+M5717**			
TAD 030	None (control)	-	-	Parental reference
TAD 377	1 × 3 mg/kg M5717	-	-	No mutations in PF3D7_1451100
TAD 382	1 × 20 mg/kg M5717	-	-	**G373S** in PF3D7_0320500, **P490L** in PF3D7_1032000, **R149T** in PF3D7_1312600, **Y186N** in PF3D7_1451100*
TAD 384	1 × 40 mg/kg M5717	-	-	**Y186N** in PF3D7_1451100*
TAD 378	1 × 20 mg/kg ZY19489 + 1 × 3 mg/kg M5717	-	-	**G373S** in PF3D7_0320500, **P490L** in PF3D7_1032000
TAD 379	1 × 20 mg/kg ZY19489 + 1 × 3 mg/kg M5717	-	-	**P490L** in PF3D7_1032000
TAD 386	1 × 20 mg/kg ZY19489 + 1 × 20 mg/kg M5717	-	-	**P490L** in PF3D7_1032000
TAD 400	1 × 20 mg/kg ZY19489 + 1 × 20 mg/kg M5717	-	-	**P490L** in PF3D7_1032000, **R149T** in PF3D7_1312600, **Y186N** in PF3D7_1451100*

a Recrudescent parasite lines were recovered from humanized NSG mice infected with the *P. falciparum* 3D7^0087/N9^ strain and treated with ZY19489, MMV693183, M5717, or ZY19489-based combinations (ZY19489+MMV693183, ZY19489+Ferroquine, or ZY19489+M5717). Parental control lines (TAD_022 and TAD_030) were sampled from untreated mice.

b IC50 values (nM) were determined for the parental controls and drug-exposed recrudescent parasites using standard 72-hour *in vitro* dose-response assays following culture adaptation. Values represent means ± SEM from 3 to 5 independent experiments, each performed in duplicate. Recrudescent parasites from the ZY19489+M5717 treatment group were not available for phenotypic analysis. Statistical significance was assessed relative to parental controls (TAD-022 or TAD_030) using two-tailed unpaired t-tests. No significant differences in susceptibility to ZY19489, MMV693183, or ferroquine were observed in any tested recrudescent line.

c Single nucleotide polymorphisms (SNPs) and copy number variations (CNVs) were identified by whole-genome sequencing of all recrudescent lines relative to the respective 3D7^0087/N9^ parental genomes (TAD-022 or TAD-030), except for TAD_377 and TAD_384 that were not whole-genome sequenced. All samples from the ZY19489+M5717 group, including TAD_377 and TAD_384, were analyzed by targeted amplicon sequencing of the *PfeEF2* (PF3D7_1451100) locus. The following genes were affected: PF3D7_0825000 (conserved *Plasmodium* protein of unknown function); PF3D7_1416100 (SEY1, a putative *Plasmodium* endoplasmic reticulum-shaping protein); PF3D7_0210200 (conserved *Plasmodium* protein of unknown function); PF3D7_1329100 (Myosin F, putative); PF3D7_1408600 (40S ribosomal protein S8e, putative); PF3D7_0320500 (nicotinamidase, putative); PF3D7_1032000 (ribosome maturation factor RimM, putative); PF3D7_1312600 (2-oxoisovalerate dehydrogenase subunit alpha, mitochondrial, putative); PF3D7_1451100 *(P. falciparum* elongation factor 2, *Pfe*EF2) - the known target of M5717. Additionally, a 3.5-fold reduction in copy number was observed in a ~1.8 kb segment of chromosome 12 harboring the *gchl* gene (PF3D7_1224000) in the TAD-496 genome. None of the observed genomic differences were associated with phenotypic resistance to ZY19489, MMV693183, or ferroquine.

* The *PfeEF2* Y186N mutation identified in M5717-exposed recrudescent lines TAD_382, TAD_384, and TAD_400 is known to confer high-grade resistance to M5717, with an approximately 16,000-fold increase in IC50.

## Data Availability

Metabolomic data is publicly accessible through the NCBI Metabolomics Workbench (Project ID: PR002514). Other datasets analyzed during the current study are provided with this paper as Supplementary Material.
